# Effect of different cytokines on mammaglobin and maspin gene expression in normal leukocytes: possible relevance to the assays for the detection of micrometastatic breast cancer

**DOI:** 10.1038/sj.bjc.6602563

**Published:** 2005-04-19

**Authors:** A Ballestrero, A Garuti, M Bertolotto, I Rocco, D Boy, A Nencioni, L Ottonello, F Patrone

**Affiliations:** 1 Department of Internal Medicine, Università di Genova, Viale Benedetto XV n. 6, 16132 Genova, Italy

**Keywords:** mammaglobin, maspin, breast cancer, micrometastasis

## Abstract

In cancer patients, the ability to detect disseminated tumour cells in peripheral blood or bone marrow could improve prognosis and consent both early detection of metastatic disease and monitoring of the efficacy of systemic therapy. These objectives remain elusive mainly due to the lack of specific genetic markers for solid tumours. The use of surrogate tissue-specific markers can reduce the specificity of the assays and give rise to a clinically unacceptable false-positive rate. Mammaglobin (MAM) and maspin are two putative breast tissue-specific markers frequently used for detection of occult tumour cells in the peripheral blood, bone marrow and lymph nodes of breast cancer patients. In this study, it was evaluated whether MAM and maspin gene expression may be induced in the normal blood and bone marrow cells exposed to a panel of cytokines, including chemotactic factors (C5a, interleukin (IL)-8), LPS, proinflammatory cytokines (TNF-*α*, IL-1*β*) and growth factors (IL-3, granulocyte-macrophage colony-stimulating factor, granulocyte colony-stimulating factor). The experimental data show that all cytokines included in the panel, except for IL-8, were able to induce maspin expression; on the contrary, MAM gene was never induced. These results suggest that MAM is more specific than maspin and that the possible interference of cytokines should be taken into account in interpreting molecular assays for detection of isolated tumour cells.

In breast cancer patients, like some other epithelial tumours, the presence of occult epithelial cells in the bone marrow or peripheral blood has been assumed as a marker of systemic malignant disease and, as a consequence, in the last two decades several immunocytochemical and molecular methods for detection of disseminated tumour cells have been developed with the aims of improving prognosis, early detection of metastatic disease or of monitoring the efficacy of systemic therapy.

Several studies suggest that these objectives can be achieved with the current assays (Cote *et al*, 1991; Harbeck *et al*, 1994; Diel *et al*, 1996; Smith *et al*, 2000; Terstappen *et al*, 2000; Gebauer *et al*, 2001; Jiang *et al*, 2002; Ozbas *et al*, 2003; Pantel *et al*, 2003) and in a few of them the presence of disseminated tumour cells in bone marrow or peripheral blood is recognised as an independent prognostic factor (Diel *et al*, 1996; Braun *et al*, 2000; Stathopoulou *et al*, 2002). However, the literature reports conflicting results and the clinical value of these assays remains to be proven basically because it is uncertain whether the published assays have enough sensitivity, specificity and consistency to be reliably integrated into prospective studies provided with adequate statistical power to answer the most relevant clinical questions (Jiang *et al*, 2002; Ozbas *et al*, 2003; Pantel *et al*, 2003). Accordingly, the most recent TNM classification of breast cancer does not consider the lymph nodes harbouring isolated tumour cells as positive, but it simply suggests reporting this information, together with the method of detection, in order to facilitate data collection (Singletary *et al*, 2002).

The molecular methods are mainly based on a qualitative reverse transcriptase polymerase chain reaction (RT-PCR) identification of breast tissue-related mRNA sequences and show, in experimental models, a sensitivity in the range of 1 : 10^6^–10^7^, which is generally higher than immunocytochemistry (Smith *et al*, 2000; Jiang *et al*, 2002).

Unlike haematological tumours, the crucial limitation of the molecular approach in breast cancer, as well as in other solid tumours, is the lack of a tumour-specific genetic marker that has induced the search for surrogate epithelial markers under the assumption that they are not expressed in mesenchymal cells. However, there are several factors such as pseudogenes, background gene expression and aberrant expression of epithelial genes in mesenchymal cells under several physiological or physiopathological conditions that can reduce the specificity of the assay and give rise to an unacceptable incidence of false-positive results (Zippelius *et al*, 1997; Jung *et al*, 1998, 1999; Ruud *et al*, 1999; Krüger *et al*, 2001; Jiang *et al*, 2002). Mammaglobin (MAM) and maspin are markers of major interest because some evidence indicates that they have a stronger relationship with breast tissue than several other epithelial markers such as cytokeratins, CEA and MUC-1 (Luppi *et al*, 1996; Zach *et al*, 1999; Grünewald *et al*, 2000; Ballestrero *et al*, 2001; Corradini *et al*, 2001; Silva *et al*, 2002; Zehentner and Carter, 2004).

Human MAM is a small epithelial secretory protein detectable in normal and pathological mammary tissue. It has a relative breast-specific expression because it is also detectable in ovary, endometrial and eccrine sweat gland tissues (Watson and Fleming, 1996; Grünewald *et al*, 2002; Sjodin *et al*, 2003).

Maspin is a serine protease inhibitor with tumour-suppressive activity due to its ability to inhibit metastatic tissue invasion, angiogenesis and to sensitise tumour cells to apoptosis (Zou *et al*, 1994; Sheng *et al*, 1996; Zhang *et al*, 2000; Cher *et al*, 2003; Liu *et al*, 2004). Maspin is also expressed in prostatic cells and other tissues (Pemberton *et al*, 1997).

Although both MAM and maspin are frequently used for detection of occult tumour cells in peripheral blood, bone marrow and lymph nodes (Luppi *et al*, 1996; Zach *et al*, 1999; Grünewald *et al*, 2000; Ballestrero *et al*, 2001; Corradini *et al*, 2001; Silva *et al*, 2002; Zehentner and Carter, 2004), their specificity has been questioned by some authors (Min *et al*, 1998; Merrie *et al*, 1999; López-Guerrero *et al*, 1999; Suchy *et al*, 2000).

Taking into account that the ectopic transcription of epithelial mRNA sequences is a decisive factor for the assay's specificity, in this article it has been evaluated whether certain biological factors may induce MAM and/or maspin expression in mesenchimal cells.

Reverse transcriptase polymerase chain reaction was used to evaluate the expression of MAM and maspin in normal blood and bone marrow cells exposed to several cytokines, chemokines and growth factors involved in the regulation of chemotaxis, inflammation and haematopoiesis.

## MATERIALS AND METHODS

### Tissue samples, cell culture and cytokine stimulation

Samples from 10 non-neoplastic mammary tissue biopsies and from 12 histologically confirmed breast cancer specimens (10 primary tumours and 2 liver metastases) were used as positive control together with the tumour cell line MCF-7 expressing both MAM and maspin genes (Luppi *et al*, 1996; Krüger *et al*, 2001). The tissue specimens were frozen in liquid nitrogen within 60 min from surgical removal and kept frozen until tissue homogenisation prior to RNA extraction.

Bone marrow specimens (3–5 ml) from 35 healthy bone marrow donors were used as negative controls to test the specificity of the RT-PCR assays. Bone marrow specimens from patients with malignant haematological disease, that is, 10 non-Hodgkin's lymphomas (NHL), five multiple myeloma (MM) and two acute myeloid leukaemias (AML), were also tested for the MAM and maspin transcript expression. Peripheral blood samples (15–20 ml) were also obtained from 35 healthy volunteers.

Polymorphonuclear (PMN) and mononuclear (Mono) cells harvested from the bone marrow and peripheral blood of 10 healthy donors were used for *in vitro* cytokine stimulation experiments.

Polymorphonuclear and Mono cells were isolated by means of dextran (Solplex 70 Società Italiana Farmaceutici, Verona, Italy) sedimentation and subsequent centrifugation on a Ficoll-Hypaque (F.H.Pharmacia, Uppsala, Sweden) density gradient. Contaminating erythrocytes were removed by hypotonic lysis. The cells were washed three times with PBS and cultured at 5 × 10^6^ ml^−1^ in RPMI 1640 with HEPES 25 mM medium (EuroClone, UK) supplemented with 10% FCS (ICN, Milano, Italy), 1% L-glutamine (200 mM, Irvine Scientific) and 1% antibiotics (penicillin 5000 U ml^−1^ and streptomycin 5000 *μ*g ml^−1^, Gibco Lifethecnology, USA).

Human recombinant cytokines, growth factors and chemotactic factors were added at the following concentration: INF-*ã* 100 U ml^−1^ (ICN, Milano, Italy), hr-tumour necrosis factor alfa (TNF-*α*) 10 ng ml^−1^ equal to 200 U ml^−1^ (ICN, Milano, Italy), hr-72 amino acids interleukin-8 (IL-8) 10 U *μ*g^−1^ 10^−7^ M (Biosource International, CA, USA), hr-interleukin-1*β* (IL-1) 10 ng ml^−1^ equal to 1000 U ml^−1^ (Biosource International, CA, USA), hr-interleukin-3 (IL-3) 10 ng ml^−1^ equal to 1000 U ml^−1^ (Biosource International, CA, USA), hr-granulocyte-macrophage colony-stimulating factor (GM-CSF) 10 ng ml^−1^ (Genzyme, Cambrige, MA, USA), hr-granulocyte colony-stimulating factor (G-CSF) 10 ng ml^−1^ (Lenograstim, Italfarmaco, Italy), LPS *Escherichia coli* 011:B4 10 *μ*g ml^−1^ (Calbiochem-Novabiochem Corporation, Darmstadt Germany), hr-Complement C5a 10^−7^ M (Sigma Chemical Co, St Louis, MO, USA).

Leukocytes were incubated with culture medium alone or cytokines for 4, 8, 20, 72 and 96 h. Each test was performed in triplicate and each sample was considered positive if at least two of the three tests were positive.

### cDNA synthesis and RT-PCR for MAM and maspin

Total RNA was extracted using Trizol solution according to the manufacturer's instructions. In all, 1 *μ*g of total RNA was reverse transcribed as described previously (Ballestrero *et al*, 2001).

Before amplification, all the cDNA samples were tested using a standard protocol to amplify a gene-specific ABL sequence as positive control (Ballestrero *et al*, 2001).

Mammaglobin RT-PCR assay was performed as follows. The external primers used were MG-1 5′-gAAgTTgCTgATggTCCTCATgCTggC-3′ and MG-2 5′-CTCACCATACCCTgCAgTTCTgTgAgC-3′. The nested primers were MG-3 5′-CTCCCAgCACTgCTACgCAggCTC-3′ and MG-4 5′-CACCTCAACATTgCTCAgAgTTTCATCCg-3′ (Zach *et al*, 1999). The lengths of the primary and reamplification products were 326 and 203 bp, respectively. The samples were subjected to 30 cycles of amplification (30 s at 95°C, 30 s at 62°C and 30 s at 72°C) and the second amplification was carried out with 3 *μ*l of the first reaction product under the same conditions as the first reaction.

Maspin nested PCR analysis was performed as reported previously (Ballestrero *et al*, 2001).

In all, 10 *μ*l of the RT-PCR reactions were electrophoresed on a 2% agarose gel and stained with ethidium bromide for visualisation under UV light.

### Statistical analysis

Confidence intervals were evaluated according to the Miettinen (1970) test.

## RESULTS

### Detection, specificity and sensitivity of MAM and maspin transcripts

Mammaglobin and maspin transcripts were detected by the nested RT-PCR method in each positive control tissue, 10 non-neoplastic mammary tissue samples, 10 primary BC and two liver metastases and the mammary tumour cell line MCF-7.

To determine the specificity of the two RT-PCR assays, specimens from both 35 bone marrow and 35 peripheral blood normal donors were evaluated.

The MAM and maspin transcripts were negative in every specimen, even after reamplification with nested primers. Thus, test specificity was 100% with a 95% confidence interval ranging from 90 to 100%, given these sample sizes.

**Figure 1:**
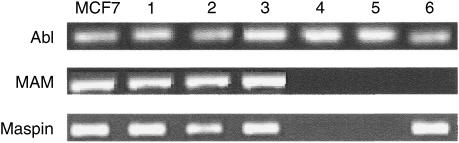
Electrophoretic analysis in a 2% agarose gel of MAM and maspin RT-PCR fragments obtained from different tissues. ABL: Abelson transcript, this sequence was used as housekeeping gene; MCF7: positive control; 1: non-neoplastic breast tissue; 2: primary breast carcinoma; 3: breast carcinoma metastases; 4: normal bone marrow; 5: normal peripheral blood; 6: pathologic bone marrow (NHL).

In 40% of the samples from the bone marrow of patients with haematological malignancies an ‘illegitimate’ maspin transcription was observed, whereas MAM transcript was undetectable (Table 1 and Figure 1).

In order to assess the sensitivity of the assay, a mixing study was performed using serial dilutions of the mammary carcinoma cell line MCF-7 in peripheral blood Mono cells from healthy volunteers. By using this approach, the nested primer RT-PCR assay was able to detect one MCF-7 cell mixed with 10^7^ normal cells, for both MAM and maspin assays according to previously published data (Luppi *et al*, 1996; Zach *et al*, 1999; Ballestrero *et al*, 2001) (data not shown).

### Effect of chemotactic factors, cytokines and growth factors on maspin and MAM gene expression

Bone marrow and peripheral leukocytes can be exposed in different physiological and physiopathological conditions to several cytokines that could induce expression of epithelial markers in the absence of true contamination by epithelial cells.

To investigate this possibility, the expression of MAM and maspin was evaluated in PMN and Mono cells exposed *in vitro* to a panel of cytokines including chemotactic factors (C5a, IL-8), LPS, pro-inflammatory cytokines (TNF-*α*, IL-1*β*) and growth factors (IL-3, GM-CSF, G-CSF).

Mammaglobin and maspin were never expressed in control samples where PMN and Mono cells were exposed to culture medium alone. Otherwise, in experiments with cytokines, all factors, with the exception of IL-8, were able to consistently induce the expression of maspin gene in either bone marrow or peripheral blood leukocytes (Table 2).

Both bone marrow PMN and Mono cells were sensitive to TNF-*α*, LPS, C5a, IL-1 and IL-3. Granulocyte colony-stimulating factor and INF-*ã* were able to stimulate bone marrow PMN and Mono cells, respectively.

The effects of cytokines on peripheral blood cells were more selective.

**Figure 2:**
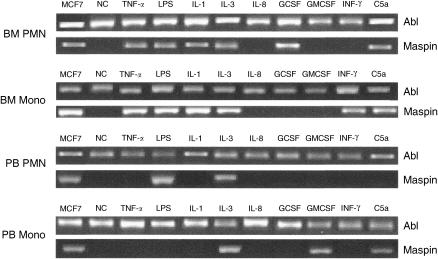
Electrophoretic analysis in a 2% agarose gel of maspin RT-PCR fragments obtained after incubation of bone marrow and peripheral blood PMN and Mono with the indicated cytokines. ABL: Abelson transcript, this sequence was used as housekeeping gene; NC: negative control, cells incubated with culture medium alone.

LPS was able to induce maspin expression in PMN, C5a and GM-CSF in Mono cells and IL-3 in both. The electrophoretic analysis of one representative experiment is reported in Figure 2.

The kinetic of expression was different between PMN and Mono cells; in fact maspin mRNA was detectable after 4 h of incubation in PMN and after 4 days in Mono cells. Interestingly, a longer incubation period, that is, 8 and 20 h, resulted in a downregulated expression of maspin by PMN.

On the contrary, the expression of MAM was never induced in PMN or Mono cells from either bone marrow or peripheral blood even when exposition to cytokines was prolonged to 4 days.

## DISCUSSION

The ability to detect the systemic disease in early-stage solid tumours has, theoretically, a sound biological foundation and could consent a rational clinical approach to the neoplastic patients in terms of both prognosis and treatment planning.

In recent years the presence of occult epithelial cells in bone marrow or peripheral blood has been assumed as a marker of systemic malignant disease in spite of the current immunocytochemical and molecular assays not giving information about the clonogenic potential of detected tumour cells. This assumption is apparently supported by some prospective studies suggesting that the presence of isolated tumour cells in bone marrow or peripheral blood is an independent prognostic factor (Diel *et al*, 1996; Braun *et al*, 2000; Stathopoulou *et al*, 2002). However, the literature reports conflicting results; so this subject is still under discussion (Jiang *et al*, 2002; Ozbas *et al*, 2003; Pantel *et al*, 2003).

Compared with immunocytochemistry and Southern or Northern blotting methods, RT-PCR assays are relatively easy and straightforward to perform, have a high sensitivity rate and are suitable for analysing large numbers of cells. However, due to the lack of tumour-specific genetic alterations, the RT-PCR-based methods rely on the amplification of surrogate epithelial markers that can give rise to false-positive results as a consequence of several physiological or physiopathological factors (Zippelius *et al*, 1997; Jung *et al*, 1998; Jung *et al*, 1999; Ruud *et al*, 1999; Krüger *et al*, 2001; Jiang *et al*, 2002).

In this study, we first compared RT-PCR detection of the two breast cancer-specific markers maspin and MAM for sensitivity and specificity. Both of the markers demonstrated similar capacities to detect isolated tumour cells (about 1 out of 10^7^), and high specificity, not producing positive results in samples obtained from healthy donors. However, subsequent testing of maspin and MAM expression in leukocyte samples from patients with haematological/inflammatory disorders indicated that maspin mRNA was expressed in 40% of these samples whereas MAM was never detectable.

Expression of epithelial markers in the bone marrow of patients with nonepithelial tumours or inflammatory diseases has previously been reported (Zippelius *et al*, 1997; Zach *et al*, 1999; Ballestrero *et al*, 2001; Krüger *et al*, 2001). Therefore, we evaluated in normal blood cells the possibility of a cytokine-induced expression of maspin and MAM.

In particular, we evaluated the effect on blood cells of a panel of known leukocyte-activating cytokines including chemotactic factors (C5a, IL-8), LPS, proinflammatory cytokines (TNF-*α*, IL-1*β*) and growth factors (IL-3, GM-CSF, G-CSF).

All of the stimuli, except for IL-8, were able to induce maspin mRNA expression in normal bone marrow PMN or Mono cells (Table 2). Conversely, only LPS, C5a, IL-3, G-CSF and GM-CSF induced maspin expression in peripheral blood cells. The reasons for the different expression pattern of maspin between bone marrow- and peripheral blood-derived cells are unclear, and may involve a reduced sensitivity to cytokine-mediated gene induction in terminally differentiated cells. Interestingly, while maspin messenger became detectable in PMN after a few hours of exposure to the activating stimuli, maspin expression in Mono cells required a prolonged stimulation. On the contrary, the MAM gene was never inducible by any of the cytokines used in the stimulation experiments.

These results provide an explanation for the detection of maspin mRNA in the peripheral blood and bone marrow samples of patients with haematological diseases and inflammation. Besides, they suggest that maspin should be considered an epithelial marker with low specificity, whereas MAM may represent a more specific marker for the detection of isolated tumour cells in peripheral blood or bone marrow samples.

However, we found that MAM expression, similar to maspin although to a lesser extent, is induced in normal leukocytes by apheretic procedures (MAM and maspin were positive, respectively, in 10 and 30% of apheretic products from normal donors with negative peripheral blood before apheretic procedure, data not shown). This observation is consistent with the previous observations by Krüger *et al* (2001), who found MAM expression in 7% of clinical samples of bone marrow and leukapheresis products obtained from patients without epithelial cancer both in basal condition and under stimulation by several cytokines, including G- and GM-CSF, IL-3, IL-1 and INF-*ã*. The reason for this effect is not understood, but since MAM is not inducible by cytokines, at least in our *in vitro* model, other mechanisms such as mechanical stress or reagents used during this procedure may play a role in this context.

This observation suggests that the apheretic procedure *per se* may be sufficient to upregulate in leukocytes markers other than maspin, through a mechanism that is possibly not cytokine-mediated. Thus, RT-PCR detection of isolated tumour cells on apheretic products may be unreliable.

In conclusion, our data suggest that MAM is more specific than maspin and should be considered a reliable epithelial marker for the detection of disseminated tumour cells in patients’ samples, probably with the exception of apheresis products. Furthermore, these data suggest that several factors possibly responsible for an ectopic gene expression should be taken into account in the validation process of new molecular markers.

## Figures and Tables

**Table 1:** Detection of MAM and maspin mRNA in breast tissues, bone marrow and peripheral blood control specimens

**Sample source**	**No. of samples**	**No. MAM positive**	**No. maspin positive**
Non-neoplastic breast tissue	10	10	10
Primary breast carcinoma	10	10	10
Breast carcinoma metastases	2	2	2
Normal bone marrow	35	0	0
Normal peripheral blood	35	0	0
Pathologic bone marrow^a^	17	0	7

a Pathologic bone marrow included 10 NHL samples, five MM samples and two AML samples.

**Table 2:** Maspin expression induced by cytokines in bone marrow and peripheral blood cells from 10 healthy volunteers

	**Neg. control**	**TNF-*α***	**LPS**	**C5a**	**IL-1**	**IL-3**	**IL-8**	**G-CSF**	**GM-CSF**	***γ* IFN**
ABL	+	+	+	+	+	+	+	+	+	+

Bone marrow										
PMN	−	+	+	+	+	+	−	+	−	−
Mono	−	+	+	+	+	+	−	−	−	+

Peripheral blood										
PMN	−	−	+	−	−	+	−	−	−	−
Mono	−	−	−	+	−	+	−	−	+	−

Positivity was defined as a minimum of seven out of 10 positive results.
